# Supplementary material for: Quantitative heterodonty in Crocodylia: assessing size and shape across modern and extinct taxa
Source: PeerJ. 2019 Feb 28;7:e6485. doi: 10.7717/peerj.6485 (PMC6397764; doi:10.7717/peerj.6485)

**Taxonomic rankings of specimens:**

**Table S1:** Taxonomic rankings and counts of specimens used in the text of this study

| **Order** | **Superfamily** | **Family** | **Subfamily** | **Species** | **N** |
| --- | --- | --- | --- | --- | --- |
| Crocodylia | Alligatoroidea | Alligatoridae | Alligatorinae | *Alligator mississippiensis* | 2 |
| Crocodylia | Alligatoroidea | Alligatoridae | Alligatorinae | *Alligator prenasalis* | 1 |
| Crocodylia | Alligatoroidea | Alligatoridae | Alligatorinae | *Alligator sinensis* | 2 |
| Crocodylia | - | - | - | *Borealosuchus sternbergii* | 2 |
| Crocodylia | Alligatoroidea | - | - | *Brachychampsa* sp. | 1 |
| Crocodylia | Alligatoroidea | Alligatoridae | Caimaninae | *Caiman crocodilus* | 3 |
| Crocodylia | Alligatoroidea | Alligatoridae | Caimaninae | *Caiman yacare* | 2 |
| Crocodylia | Crocodyloidea | Crocodylidae | Crocodylinae | *Crocodylus acutus* | 2 |
| Crocodylia | Crocodyloidea | - | - | *"Crocodylus" affinis* | 1 |
| Crocodylia | Crocodyloidea | Crocodylidae | Crocodylinae | *Crocodylus niloticus* | 2 |
| Crocodylia | Crocodyloidea | Crocodylidae | Crocodylinae | *Crocodylus palustris* | 2 |
| Crocodylia | Crocodyloidea | Crocodylidae | Crocodylinae | *Crocodylus porosus* | 2 |
| Crocodylia | Crocodyloidea | Crocodylidae | Crocodylinae | *Crocodylus siamensis* | 2 |
| Crocodylia | Gavialoidea | Gavialidae | Gavialinae | *Gavialis gangeticus* | 1 |
| - | - | Peirosauridae | - | *Hamadasuchus rebouli* | 1 |
| Crocodylia | Alligatoroidea | - | - | *Leidyosuchus canadensis* | 1 |
| Crocodylia | Crocodyloidea | Crocodylidae | Crocodylinae | *Mecistops cataphractus* | 1 |
| Crocodylia | Crocodyloidea | Crocodylidae | Crocodylinae | *Osteolaemus tetraspis* | 2 |
| Crocodylia | Alligatoroidea | Alligatoridae | Caimaninae | *Paleosuchus palpebrosus* | 2 |
| Crocodylia | Alligatoroidea | Alligatoridae | Caimaninae | *Paleosuchus trigonatus* | 2 |
| Crocodylia | Crocodyloidea | Crocodylidae | Tomistominae | *Tomistoma schlegelii* | 1 |
|  |  |  |  | **Total** | **35** |

**Specimen information and regression coefficients:**

**Table S2:** Completeness of tooth row, rearing condition, and regression information, coefficients, and significance for Principal Component 1 plotted against tooth position for each crocodylian specimen. N=the ratio of tooth positions measured out of all positions based on alveoli, M=slope, B=*y*-intercept, R^2^= goodness of fit, P=significance, 95%=upper and lower bounds of 95% confidence intervals.

| **Species** | **Specimen #** | **Rearing condition** | **Host bone** | **N** | **M** | **B** | **R^2^** | **P** | **95%** | |
| --- | --- | --- | --- | --- | --- | --- | --- | --- | --- | --- |
| *Alligator mississippiensis* | ROM 4408 | No Data | Cranium | 17:20 | 0.4042 | 0.0295 | 0.8283 | <0.0001 | 0.30 | 0.51 |
|  |  |  | Dentary | 14:20 | 0.5206 | 0.0808 | 0.8099 | <0.0001 | 0.36 | 0.68 |
| *Alligator mississippiensis* | AMNH 71621 | No Data | Cranium | 17:20 | 0.3966 | 0.0471 | 0.8342 | <0.0001 | 0.30 | 0.49 |
|  |  |  | Dentary | 18:20 | 0.4495 | 0.0533 | 0.8777 | <0.0001 | 0.36 | 0.54 |
| *Alligator prenasalis* | ROM 1375 | Fossil | Cranium | 8:20 | 0.3012 | 0.2107 | 0.9092 | 0.0002 | 0.21 | 0.40 |
| *Alligator sinensis* | AMNH 23900 | Wild | Cranium | 15:19 | 0.4613 | 0.0756 | 0.8375 | <0.0001 | 0.34 | 0.58 |
|  |  |  | Dentary | 14:19 | 0.4276 | 0.0764 | 0.8901 | <0.0001 | 0.33 | 0.52 |
| *Alligator sinensis* | AMNH 23907 | Wild | Cranium | 15:19 | 0.4318 | 0.0536 | 0.8704 | <0.0001 | 0.33 | 0.53 |
|  |  |  | Dentary | 15:19 | 0.3379 | 0.0889 | 0.8116 | <0.0001 | 0.24 | 0.44 |
| *Borealosuchus sternbergii* | UCMP 126099 | Fossil | Cranium | 7:23 | 0.5478 | -0.0829 | 0.8883 | 0.0015 | 0.32 | 0.77 |
| *Borealosuchus sternbergii* | UCMP 131769 | Fossil | Dentary | 11:20 | 0.3768 | -0.0050 | 0.7942 | 0.0002 | 0.23 | 0.52 |
| *Brachychampsa* sp. | ROM 68491 | Fossil | Cranium | 12:19 | 0.3218 | 0.1987 | 0.9063 | <0.0001 | 0.25 | 0.39 |
|  |  |  | Dentary | 7:20 | 0.2200 | 0.2389 | 0.8718 | 0.0021 | 0.12 | 0.32 |
| *Caiman crocodilus* | AMNH 7714 | No Data | Cranium | 16:20 | 0.4502 | -0.0483 | 0.7989 | <0.0001 | 0.32 | 0.58 |
|  |  |  | Dentary | 15:20 | 0.4062 | -0.0218 | 0.8181 | <0.0001 | 0.29 | 0.52 |
| *Caiman crocodilus* | AMNH 7719 | No Data | Cranium | 18:20 | 0.4759 | -0.0229 | 0.8455 | <0.0001 | 0.37 | 0.58 |
|  |  |  | Dentary | 18:20 | 0.4231 | -0.0254 | 0.9029 | <0.0001 | 0.35 | 0.50 |
| *Caiman crocodilus* | UCMP 42844 | Fossil | Cranium | 7:20 | 0.3238 | -0.0389 | 0.7262 | 0.0149 | 0.10 | 0.55 |
|  |  |  | Dentary | 14:20 | 0.4260 | -0.0095 | 0.8453 | <0.0001 | 0.31 | 0.54 |
| *Caiman yacare* | AMNH 97297 | Wild | Cranium | 13:20 | 0.5516 | -0.0154 | 0.8744 | <0.0001 | 0.41 | 0.69 |
|  |  |  | Dentary | 15:20 | 0.4761 | -0.0029 | 0.9575 | <0.0001 | 0.42 | 0.54 |
| *Caiman yacare* | AMNH 97300 | Wild | Cranium | 14:20 | 0.4972 | -0.0562 | 0.8335 | <0.0001 | 0.36 | 0.64 |
|  |  |  | Dentary | 16:20 | 0.4428 | -0.0145 | 0.8368 | <0.0001 | 0.33 | 0.55 |
| *Crocodylus acutus* | AMNH 7856 | No Data | Cranium | 15:18 | 0.4348 | -0.0489 | 0.8742 | <0.0001 | 0.34 | 0.53 |
|  |  |  | Dentary | 11:15 | 0.4225 | -0.0443 | 0.8964 | <0.0001 | 0.31 | 0.53 |
| *Crocodylus acutus* | AMNH 7857 | No Data | Cranium | 10:18 | 0.4301 | -0.0552 | 0.8030 | 0.0004 | 0.26 | 0.60 |
|  |  |  | Dentary | 13:15 | 0.4455 | -0.0221 | 0.8894 | <0.0001 | 0.34 | 0.55 |
| *"Crocodylus" affinis* | UCMP 131090 | Fossil | Cranium | 11:19 | 0.2871 | 0.1568 | 0.9423 | <0.0001 | 0.23 | 0.34 |
| *Crocodylus niloticus* | AMNH 23471 | Wild | Cranium | 16:19 | 0.3303 | 0.0023 | 0.8089 | <0.0001 | 0.24 | 0.42 |
|  |  |  | Dentary | 15:15 | 0.3571 | -0.0351 | 0.8406 | <0.0001 | 0.26 | 0.45 |
| **Species** | **Specimen #** | **Rearing condition** | **Host bone** | **N** | **M** | **B** | **R^2^** | **P** | **95%** | |
| *Crocodylus niloticus* | AMNH 142494 | No Data | Cranium | 11:19 | 0.3694 | 0.0765 | 0.7951 | 0.0002 | 0.23 | 0.51 |
|  |  |  | Dentary | 11:15 | 0.4910 | -0.0163 | 0.8823 | <0.0001 | 0.36 | 0.63 |
| *Crocodylus palustris* | AMNH 75707 | No Data | Cranium | 9:19 | 0.3504 | -0.0202 | 0.9086 | 0.0001 | 0.25 | 0.45 |
|  |  |  | Dentary | 12:15 | 0.4897 | 0.0189 | 0.9278 | <0.0001 | 0.39 | 0.59 |
| *Crocodylus palustris* | AMNH 96134 | Wild | Cranium | 14:19 | 0.4845 | -0.0180 | 0.8334 | <0.0001 | 0.35 | 0.62 |
|  |  |  | Dentary | 9:15 | 0.3841 | -0.0071 | 0.8926 | 0.0001 | 0.27 | 0.50 |
| *Crocodylus porosus* | AMNH 66639 | No Data | Cranium | 11:19 | 0.4290 | -0.0967 | 0.8466 | 0.0001 | 0.29 | 0.14 |
|  |  |  | Dentary | 10:15 | 0.3795 | -0.1047 | 0.8522 | 0.0001 | 0.25 | 0.13 |
| *Crocodylus porosus* | AMNH 94957 | No Data | Cranium | 15:19 | 0.4359 | -0.0139 | 0.8830 | <0.0001 | 0.34 | 0.10 |
|  |  |  | Dentary | 9:15 | 0.3021 | -0.0355 | 0.7991 | 0.0012 | 0.17 | 0.14 |
| *Crocodylus siamensis* | AMNH 49231 | Wild | Cranium | 8:19 | 0.6681 | -0.0467 | 0.8998 | 0.0003 | 0.45 | 0.22 |
|  |  |  | Dentary | 12:15 | 0.5564 | -0.0594 | 0.8797 | <0.0001 | 0.41 | 0.15 |
| *Crocodylus siamensis* | AMNH 72640 | No Data | Cranium | 16:19 | 0.6828 | -0.0419 | 0.8129 | <0.0001 | 0.50 | 0.19 |
|  |  |  | Dentary | 13:15 | 0.3861 | -0.0364 | 0.8900 | <0.0001 | 0.30 | 0.09 |
| *Gavialis gangeticus* | AMNH 131377 | No Data | Cranium | 22:28 | 0.1371 | -0.2773 | 0.4952 | 0.0003 | 0.07 | 0.06 |
|  |  |  | Dentary | 24:26 | 0.2540 | -0.2464 | 0.6156 | <0.0001 | 0.17 | 0.09 |
| *Hamadasuchus rebouli* | ROM 52620 | Fossil | Cranium | 14:20 | 0.5260 | -0.0223 | 0.9211 | <0.0001 | 0.43 | 0.10 |
| *Leidyosuchus canadensis* | ROM 1903 | Fossil | Cranium | 18:23 | 0.4470 | 0.0088 | 0.9466 | <0.0001 | 0.39 | 0.06 |
| *Mecistops cataphractus* | AMNH 107634 | Wild | Cranium | 15:18 | 0.3845 | -0.1605 | 0.8504 | <0.0001 | 0.29 | 0.10 |
|  |  |  | Dentary | 15:15 | 0.3162 | -0.1427 | 0.9346 | <0.0001 | 0.27 | 0.05 |
| *Osteolaemus tetraspis* | AMNH 117801 | No Data | Cranium | 12:17 | 0.5670 | -0.0310 | 0.8703 | <0.0001 | 0.41 | 0.72 |
|  |  |  | Dentary | 11:14 | 0.4916 | 0.0169 | 0.8085 | 0.0002 | 0.31 | 0.18 |
| *Osteolaemus tetraspis* | AMNH 117802 | No Data | Cranium | 15:17 | 0.5499 | 0.0061 | 0.7599 | <0.0001 | 0.36 | 0.19 |
|  |  |  | Dentary | 14:14 | 0.5118 | 0.0319 | 0.8559 | <0.0001 | 0.38 | 0.13 |
| *Paleosuchus palpebrosus* | AMNH 93812 | No Data | Cranium | 19:20 | 0.4883 | -0.0123 | 0.8731 | <0.0001 | 0.39 | 0.10 |
|  |  |  | Dentary | 20:22 | 0.3930 | 0.0175 | 0.9028 | <0.0001 | 0.33 | 0.06 |
| *Paleosuchus palpebrosus* | AMNH 97328 | Wild | Cranium | 18:20 | 0.4905 | -0.0282 | 0.8647 | <0.0001 | 0.39 | 0.10 |
|  |  |  | Dentary | 13:22 | 0.5093 | 0.0070 | 0.8989 | <0.0001 | 0.40 | 0.11 |
| *Paleosuchus trigonatus* | AMNH 58136 | Wild | Cranium | 16:20 | 0.6097 | -0.0753 | 0.9017 | <0.0001 | 0.49 | 0.12 |
|  |  |  | Dentary | 20:22 | 0.4335 | 0.0436 | 0.8348 | <0.0001 | 0.34 | 0.10 |
| *Paleosuchus trigonatus* | AMNH 137174 | No Data | Cranium | 14:20 | 0.5379 | -0.0688 | 0.8434 | <0.0001 | 0.39 | 0.15 |
|  |  |  | Dentary | 14:22 | 0.3975 | -0.0092 | 0.8238 | <0.0001 | 0.28 | 0.12 |
| *Tomistoma schlegelii* | AMNH 113078 | No Data | Cranium | 13:21 | 0.3912 | -0.1797 | 0.7474 | 0.0001 | 0.24 | 0.15 |
|  |  |  | Dentary | 18:19 | 0.3438 | -0.1754 | 0.8479 | <0.0001 | 0.27 | 0.08 |

**Principal components:**

We only included principal components (PCs) that accounted for over 5% of the shape variance to compare to position (Table S3). PC2 and PC3 were further analyzed here to determine if they were biological relevant concerning heterodonty. As stated in the main text, PC2 is the orientation of the tooth, or how much it ‘leans’ (Figure 2). PC3 is the degree to which to tooth tapers in proximity of the apex (Figure S1). Positive values indicate a tapered tooth with a ‘sharp’ apex, and negative values indicate a ‘blunt’ tooth with a rounded apex.

 In order to determine if PC2 or PC3 had any relevance to heterodonty, an ordinary least squares regression analysis was conducted. For all teeth, the first three PCs were plotted against position to determine significance and regression coefficients. Position was a continuous variable, and converted into a percentage. We numbered the positions along the tooth row starting with 1 at the mesial-most position, divided each by the total number of positions along the arcade, and then subtracted 0.5 (see Materials and Methods for further explanation). PC1 (y=-0.426x-0.020; r^2^=0.560; *p*<0.0001; 95% =0.402, 0.450) was significantly correlated to position, but PC2 (y=-0.004x+0.000; r^2^=0.002; *p*=0.2132; 95%=-0.012, 0.003) and PC3 (y=-0.003x+0.000; r^2^=0.001; *p*=0.3281; 95%=-0.003, 0.008) were not.

 There are potential explanations for the shape variance represented by PC2 and PC3. PC2 could be developmental, and reflect the tooth growing unevenly. This may cause the apex to lean in a certain direction, as this direction may result in one margin that is shorter than the other. This could also be researcher error. Although we designate it as a repeatable measurement, the point where the neck ceased to taper may not have been clear in all teeth, resulting in an artificially long or short margin on either side of the tooth. PC3 is most likely a consequence of wear. Although teeth with large wear facets were excluded, minor wear was included. Variability in the degree to which the apices are rounded could be a consequence of this. We do not know how age of the tooth and/or the nature of replacement would affect wear, but it appears to not relate to tooth position in a linear fashion.

**Table S3:** Principal Components analysis of all teeth in the sample. “% Variance” represents the proportion of the overall variance represented by each component.

| **Principal Component** | **Eigen- value** | **% Variance** | **Principal Component** | **Eigen-value** | **% Variance** | **Principal Component** | **Eigen- value** | **% Variance** |
| --- | --- | --- | --- | --- | --- | --- | --- | --- |
| 1 | 0.026 | 92.107 | 39 | <0.001 | 0.001 | 77 | <0.001 | <0.001 |
| 2 | 0.001 | 3.222 | 40 | <0.001 | 0.001 | 78 | <0.001 | <0.001 |
| 3 | <0.001 | 1.689 | 41 | <0.001 | 0.001 | 79 | <0.001 | <0.001 |
| 4 | <0.001 | 1.070 | 42 | <0.001 | 0.001 | 80 | <0.001 | <0.001 |
| 5 | <0.001 | 0.619 | 43 | <0.001 | 0.001 | 81 | <0.001 | <0.001 |
| 6 | <0.001 | 0.454 | 44 | <0.001 | 0.001 | 82 | <0.001 | <0.001 |
| 7 | <0.001 | 0.216 | 45 | <0.001 | 0.001 | 83 | <0.001 | <0.001 |
| 8 | <0.001 | 0.159 | 46 | <0.001 | 0.001 | 84 | <0.001 | <0.001 |
| 9 | <0.001 | 0.107 | 47 | <0.001 | 0.001 | 85 | <0.001 | <0.001 |
| 10 | <0.001 | 0.069 | 48 | <0.001 | 0.001 | 86 | <0.001 | <0.001 |
| 11 | <0.001 | 0.042 | 49 | <0.001 | 0.001 | 87 | <0.001 | <0.001 |
| 12 | <0.001 | 0.035 | 50 | <0.001 | 0.001 | 88 | <0.001 | <0.001 |
| 13 | <0.001 | 0.029 | 51 | <0.001 | 0.001 | 89 | <0.001 | <0.001 |
| 14 | <0.001 | 0.026 | 52 | <0.001 | 0.001 | 90 | <0.001 | <0.001 |
| 15 | <0.001 | 0.021 | 53 | <0.001 | 0.001 | 91 | <0.001 | <0.001 |
| 16 | <0.001 | 0.014 | 54 | <0.001 | 0.001 | 92 | <0.001 | <0.001 |
| 17 | <0.001 | 0.013 | 55 | <0.001 | 0.001 | 93 | <0.001 | <0.001 |
| 18 | <0.001 | 0.010 | 56 | <0.001 | 0.001 | 94 | <0.001 | <0.001 |
| 19 | <0.001 | 0.009 | 57 | <0.001 | 0.001 | 95 | <0.001 | <0.001 |
| 20 | <0.001 | 0.008 | 58 | <0.001 | 0.001 | 96 | <0.001 | <0.001 |
| 21 | <0.001 | 0.007 | 59 | <0.001 | 0.001 | 97 | <0.001 | <0.001 |
| 22 | <0.001 | 0.005 | 60 | <0.001 | 0.001 | 98 | <0.001 | <0.001 |
| 23 | <0.001 | 0.005 | 61 | <0.001 | 0.001 | 99 | <0.001 | <0.001 |
| 24 | <0.001 | 0.005 | 62 | <0.001 | 0.001 | 100 | <0.001 | <0.001 |
| 25 | <0.001 | 0.004 | 63 | <0.001 | 0.001 | 101 | <0.001 | <0.001 |
| 26 | <0.001 | 0.004 | 64 | <0.001 | 0.001 | 102 | <0.001 | <0.001 |
| 27 | <0.001 | 0.003 | 65 | <0.001 | <0.001 | 103 | <0.001 | <0.001 |
| 28 | <0.001 | 0.003 | 66 | <0.001 | <0.001 | 104 | <0.001 | <0.001 |
| 29 | <0.001 | 0.003 | 67 | <0.001 | <0.001 | 105 | <0.001 | <0.001 |
| 30 | <0.001 | 0.002 | 68 | <0.001 | <0.001 | 106 | <0.001 | <0.001 |
| 31 | <0.001 | 0.002 | 69 | <0.001 | <0.001 | 107 | <0.001 | <0.001 |
| 32 | <0.001 | 0.002 | 70 | <0.001 | <0.001 | 108 | <0.001 | <0.001 |
| 33 | <0.001 | 0.002 | 71 | <0.001 | <0.001 | 109 | <0.001 | <0.001 |
| 34 | <0.001 | 0.002 | 72 | <0.001 | <0.001 | 110 | <0.001 | <0.001 |
| 35 | <0.001 | 0.002 | 73 | <0.001 | <0.001 | 111 | <0.001 | <0.001 |
| 36 | <0.001 | 0.002 | 74 | <0.001 | <0.001 | 112 | <0.001 | <0.001 |
| 37 | <0.001 | 0.001 | 75 | <0.001 | <0.001 | 113 | <0.001 | <0.001 |
| 38 | <0.001 | 0.001 | 76 | <0.001 | <0.001 | 114 | <0.001 | <0.001 |

**Figure S1: Variability within the third principal components for teeth.** Vector diagrams indicate the maximum range of variance (vectors) from the mean (points) for both cranial and dentary teeth. Landmarks are in magenta and semilandmarks are in green.


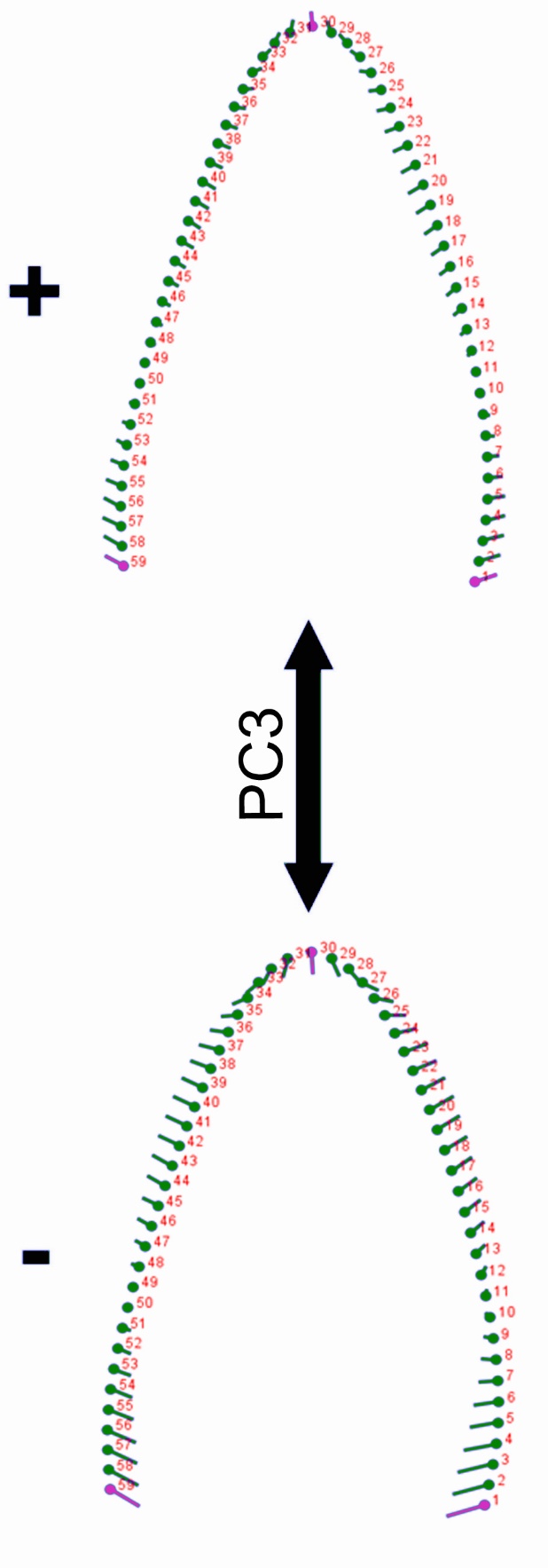

Supplement: Supplemental Information 1 — A PDF document of taxonomic rankings of organisms sampled, an explanation of the Principal Components not used in the study, tables of PC eigenvalues and variances, detailed regression information for shape heterodonty, and an analysis and discussion on the potential effects of captive rearing. The file includes text, tables, and figures. [file peerj-07-6485-s001.docx]
